# Plasma amyloid-β levels, cerebral atrophy and risk of dementia: a population-based study

**DOI:** 10.1186/s13195-018-0395-6

**Published:** 2018-06-30

**Authors:** Saima Hilal, Frank J. Wolters, Marcel M. Verbeek, Hugo Vanderstichele, M. Kamran Ikram, Erik Stoops, M. Arfan Ikram, Meike W. Vernooij

**Affiliations:** 1 Department of Radiology and Nuclear Medicine, Erasmus University Medical Center, Office no. 2505, Wytemaweg 80, 3015 CN Rotterdam, The Netherlands; 2 Department of Epidemiology, Erasmus University Medical Center, Rotterdam, The Netherlands; 3 Department of Neurology and Laboratory Medicine, Donders Institute for Brain, Cognition and Behavior, Radboud Alzheimer Centre, Radboud University Medical Center, Nijmegen, The Netherlands; 4 ADx NeuroSciences, Ghent, Belgium; 5 Department of Neurology, Erasmus University Medical Center, Rotterdam, The Netherlands

**Keywords:** Plasma amyloid-β levels, Magnetic resonance imaging, Atrophy, Dementia, Population-based

## Abstract

**Background:**

Plasma amyloid-β (Aβ) levels are increasingly studied as a potential accessible marker of cognitive impairment and dementia. However, it remains underexplored whether plasma Aβ levels including the novel Aβ peptide 1–38 (Aβ_1–38_) relate to preclinical markers of neurodegeneration and risk of dementia. We investigated the association of plasma Aβ_1–38_, Aβ_1–40_, and Aβ_1–42_ levels with imaging markers of neurodegeneration and risk of dementia in a prospective population-based study.

**Methods:**

We analyzed plasma Aβ levels in 458 individuals from the Rotterdam Study. Brain volumes, including gray matter, white matter, and hippocampus, were computed on the basis of 1.5-T magnetic resonance imaging (MRI). Dementia and its subtypes were defined on the basis of internationally accepted criteria.

**Results:**

A total of 458 individuals (mean age, 67.8 ± 7.7 yr; 232 [50.7%] women) with baseline MRI scans and incident dementia were included. The mean ± SD values of Aβ_1–38_, Aβ_1–40_, and Aβ_1–42_ (in pg/ml) were 19.4 ± 4.3, 186.1 ± 35.9, and 56.3 ± 6.2, respectively, at baseline. Lower plasma Aβ_1–42_ levels were associated with smaller hippocampal volume (mean difference in hippocampal volume per SD decrease in Aβ_1–42_ levels, − 0.13; 95% CI, − 0.23 to − 0.04; *p* = 0.007). After a mean follow-up of 14.8 years (SD, 4.9; range, 4.1–23.5 yr), 79 persons developed dementia, 64 of whom were diagnosed with Alzheimer’s disease (AD). Lower levels of Aβ_1–38_ and Aβ_1–42_ were associated with increased risk of dementia, specifically AD (HR for AD per SD decrease in Aβ_1–38_ levels, 1.39; 95% CI, 1.00–2.16; HR for AD per SD decrease in Aβ_1–42_ levels, 1.35; 95% CI, 1.05–1.75) after adjustment for age, sex, education, cardiovascular risk factors, apolipoprotein E ε4 allele carrier status, and other Aβ isoforms.

**Conclusions:**

Our results show that lower plasma Aβ levels were associated with risk of dementia and incident AD. Moreover, lower plasma Aβ_1–42_ levels were related to smaller hippocampal volume. These results suggest that plasma Aβ_1–38_ and Aβ_1–42_ maybe useful biomarkers for identification of individuals at risk of dementia.

## Background

Dementia due to Alzheimer’s disease (AD), constituting up to 70% of all dementias, is characterized by deposition of amyloid plaques and neurofibrillary tangles in the brain parenchyma [1]. Amyloid-β (Aβ) 1–40 (Aβ_1–40_) and Aβ_1–42_ peptides derived from amyloid precursor protein are present in these parenchymal plaques, and it is hypothesized that Aβ peptide abnormalities begin early in the neurodegenerative pathological cascade [2, 3]. According to this amyloid cascade hypothesis, an imbalance between the production and clearance of the Aβ peptides, especially the Aβ_1–42_ isoform, leads to their aggregation in the cortical tissue and vessels [3]. This Aβ deposition in the brain subsequently affects plasma concentrations, thus suggesting that circulating levels of Aβ could potentially be used as markers of disease risk [4].

Aβ_1–40_ and Aβ_1–42_ peptides are the two most studied AD biomarkers, which can be measured in plasma through minimally invasive techniques and thus can feasibly be determined in large samples. Several large community-based studies have reported on the association of high plasma Aβ_1–40_ and Aβ_1–42_ levels and lower Aβ_1–42_/Aβ_1–40_ ratios at baseline with risk of dementia and reduced cognitive measure scores [5–7]. Two studies have shown a link between higher levels of Aβ_1–42_ but not Aβ_1–40_ with risk of AD [8, 9]. Conversely, some studies reported an association of increased concentrations of Aβ_1–42_ and Aβ_1–40_ levels and higher Aβ_1–42_/Aβ_1–40_ ratio with reduced risk of dementia [10, 11]. Longitudinal studies with repeated measurements of Aβ have shown that individuals in preclinical stages of the disease [12] and persons with newly diagnosed AD [6] showed significant reductions in plasma Aβ_1–42_ concentrations over time. Though most of the study results are inconsistent, they nevertheless do suggest that plasma Aβ levels may be differentially associated with the risk for AD, possibly reflective of the stage of the disease and the complex pattern of production and clearance from the brain [4]. Limited data also suggest that increased circulating levels of Aβ_1–40_ and Aβ_1–42_ may induce cerebral atrophy, detected on magnetic resonance imaging (MRI) studies as cortical thinning in asymptomatic elderly persons through mechanisms such as synaptic deficits and neuronal loss [13-15] . More studies of a general population are needed to investigate the relationship between Aβ and markers of neurodegeneration as well as risk of dementia to further comprehend the underlying mechanisms in a subclinical phase.

Besides Aβ_1–40_ and Aβ_1–42_ peptides, which have been investigated extensively, another isoform—Aβ_1–38_—is of interest because it is reported to be elevated in the cerebrospinal fluid (CSF) of patients with sporadic AD [16]. Moreover, it is suggested to be a sensitive and specific marker for diagnosing AD over the range of other non-AD dementias, and subsequent studies confirmed the suitability of Aβ_1–38_ as an additional biomarker for differential diagnosis of dementia [17, 18]. Despite this ongoing research, little is known regarding whether plasma Aβ levels, including the novel Aβ_1–38_, are associated with preclinical markers of neurodegeneration, such as gray matter, white matter, and hippocampal atrophy, and the risk of dementia in a large population-based setting. We examined the association of plasma Aβ_1–38_, Aβ_1–40_, and Aβ_1–42_ levels with neurodegenerative markers and risk of dementia in a subsample of the Rotterdam Study.

## Methods

### Study population

The Rotterdam Study is a population-based prospective cohort study of middle-aged and elderly persons living in the Ommoord district in the city of Rotterdam, the Netherlands. All participants in this study undergo reexamination every 3–4 years and are being followed continuously for events, including occurrence of dementia. This study is embedded within the first cohort, which was initiated in 1990 with 7983 participants at baseline (aged ≥ 55 yr). Persons were randomly selected (*n* = 563) during the second visit of the first cohort (1995–1996) and were invited to undergo neuroimaging [19]. Blood samples of the same individuals were drawn in the 1998 and 1999. After individuals with insufficient plasma (*n* = 22), no MRI scans (*n* = 73), and prevalent dementia (*n* = 10) were removed, the final sample size consisted of 458 persons [20].

### Plasma assessment

Blood samples were drawn into ethylenediaminetetraacetic acid (EDTA) tubes for plasma collection. After centrifugation (2500 × *g*, + 4 °C for 20 min), plasma samples were stored at − 80 °C within 60 minutes of collection. Plasma levels of Aβ_1–38_, Aβ_1–40_, and Aβ_1–42_ were quantified by EUROIMMUN β-Amyloid 1–38, 1–42, and 1–40 plasma enzyme-linked immunosorbent assays (EUROIMMUN, Lübeck, Germany), which have been validated and described in more detail previously [21, 22]. For quality control (QC) purposes, QC samples were produced by pooling of EDTA plasma samples from individual participants. After aliquoting, samples were stored at − 80 °C. The samples were coded QC1, QC2, QC8, QC9, and QC10 and were used in the three plasma amyloid assays. QC in the three assays was ± 2 SD of each amyloid concentration (in pg/ml) across study participants, with the ranges of concentration values detectable being 5.9–18.7 pg/ml for Aβ_1–38_, 67.6–161.8 pg/ml for Aβ_1–40_, and 46.6–55.6 pg/ml for Aβ_1–42_. The average coefficients of variation of measurement of Aβ_1–38_, Aβ_1–40_, and Aβ_1–42_ in QC plasma samples during the study were 11.04%, 5.72%, and 8.70%, respectively [22].

### Brain imaging

Brain MRI was performed using a 1.5-T MRI system (VISION MR; Siemens AG, Erlangen, Germany) to obtain T1-weighted, proton density, T2-weighted, and high-resolution inversion recovery double-contrast three-dimensional half-Fourier-acquired single-shot turbo spin echo (HASTE) sequences [23]. Image preprocessing and the tissue classification algorithm have been described elsewhere [23]. Briefly, the *k*-nearest neighbors brain tissue classifier technique was used to classify voxels into CSF, gray matter, normal white matter, and white matter hyperintensities. Intracranial volume was the sum of the CSF, gray matter, normal white matter, and white matter hyperintensities. We used a validated nonrigid registration algorithm to map brain regions to the template scan. Hippocampal volumes were manually outlined on coronal HASTE sequences perpendicular to the long axis of the hippocampus [24].

### Assessment of dementia

Participants were screened for dementia at baseline and at follow-up examinations [20]. Screening was performed using the Mini Mental State Examination (MMSE) and the Geriatric Mental State Schedule (GMS) organic level. Individuals with screen-positive results (MMSE < 26 or GMS organic level > 0) subsequently underwent an examination and informant interview with the Cambridge Examination for Mental Disorders of the Elderly. Additionally, the whole cohort was continuously monitored for dementia through computerized linkage of the study database and digitized medical records of general practitioners and the Regional Institute for Outpatient Mental Health Care. When required and available, neuroimaging was used to facilitate dementia diagnosis. A consensus panel led by a consultant neurologist established the final diagnosis according to standard criteria for dementia (*Diagnostic and Statistical Manual of Mental Disorders, Third Edition–Revised*). The diagnosis of AD was made using the National Institute of Neurological and Communicative Disorders and Stroke-Alzheimer’s Disease and Related Disorders Association [25] and National Institute of Neurological Disorders and Stroke-Association Internationale pour la Recherché et l’Enseignement en Neurosciences criteria for vascular dementia [26]. The first cohort was followed for dementia until 15 years (based on maximum follow-up) after baseline examination (i.e., January 2015). Follow-up for dementia was complete for 99.5% of potential person-years in this cohort.

### Covariate assessment

Data on demographics and medical history were recorded on the same day of dementia screening. Blood pressure was measured in two readings using a random zero sphygmomanometer in a sitting position, and the mean of both measurements was calculated. Mean arterial blood pressure was calculated as two-thirds of the diastolic blood pressure plus one-third of the systolic blood pressure. Serum total cholesterol levels were measured using an automated enzymatic procedure. Diabetes mellitus was defined as fasting blood glucose ≥ 7 mmol/L or receiving treatment for diabetes. Smoking was categorized into ever versus never smokers. Education was treated as the number of years of formal education. Apolipoprotein E (APOE) genotype was determined using PCRs on coded DNA samples. Distribution of APOE genotype and allele frequencies were in Hardy-Weinberg equilibrium. APOE-ε4 carrier status was defined by the presence of at least one ε4 allele.

### Statistical analysis

Plasma Aβ levels and brain tissue volumes were standardized (by subtracting each variable by population mean divided by SD). Plasma Aβ levels were expressed as per-SD decrease. The Aβ_1–40_/Aβ_1–42_ ratio was calculated using raw values of Aβ_1–40_ and Aβ_1–42_ levels. We first performed linear regression models to determine the association between Aβ levels and brain tissue volumes (total brain volumes, white matter volume, gray matter volume, and hippocampal volume). The models were adjusted for age, sex, intracranial volume, mean arterial blood pressure, total cholesterol, diabetes, smoking, and APOE-ε4 carrier status. We tested the independent effects of Aβ levels with brain tissue volumes by adding all three isoforms together in the regression models.

Using Cox proportional hazards models, we calculated HRs with corresponding 95% CIs for dementia and its subtypes with per-SD decrease in Aβ level. Participants were censored within the follow-up period at date of event diagnosis, death, or loss to follow-up, whichever came first. The proportional hazards assumption was tested by adding the interaction terms of Aβ_1–38_, Aβ_1–40_, Aβ_1–42_, and Aβ_1–40_/Aβ_1–42_ ratio with follow-up time in different models. All Cox proportional hazards models were initially adjusted for age, sex, and education and subsequently for mean arterial blood pressure, total cholesterol, diabetes, smoking, APOE-ε4 carrier status, and the other Aβ isoforms using a similar approach to the one described above. We also investigated whether the association between plasma Aβ and dementia was different in carriers and noncarriers of the APOE-ε4 allele. The level of significance was set to 5%, and all tests were two-sided. Statistical analyses were performed using IBM SPSS Statistics version 24 software (IBM, Armonk, NY, USA).

## Results

Baseline characteristics of the participants are shown in Table 1. The mean age of the participants was 67.8 ± 7.7 years, and 232 (50.7%) were women. Diabetes was present in 55 (12.1%) of the study population, whereas the frequency of ever smokers was 317 (69.7%). Almost 30% (*n* = 137) persons were APOE-ε4 carriers. The mean ± SD values of Aβ_1–38_, Aβ_1–40_, and Aβ_1–42_ were 19.4 ± 4.3, 186.1 ± 35.9, and 56.3 ± 6.2, whereas for white matter volume, gray matter volume, and hippocampal volume, the respective values were 354.1 ± 85.4, 522.7 ± 55.8, and 6.4 ± 0.9. The correlation between Aβ_1–38_ and Aβ_1–40_ was 0.81 (Pearson’s correlation coefficient, *R*); between Aβ_1–38_ and Aβ_1–42_, *R* = 0.24, and between Aβ_1–40_ and Aβ_1–42_, *R* = 0.25.

**Table 1:** Baseline characteristics of the study population

Variables	Subsample of Rotterdam Study (*n* = 458)
Demographic and vascular risk factors	
Age, yr, mean (SD)	67.8 (7.7)
Women, *n* (%)	232 (50.7)
Education, yr, mean (SD)	10.7 (3.4)
Mean arterial blood pressure, mmHg, mean (SD)	96.5 (12.7)
Total cholesterol, mmol/L, mean (SD)	5.7 (0.93)
Diabetes mellitus, *n* (%)	55 (12.1)
Smoker, ever, *n* (%)	317 (69.7)
APOE-ε4 carriers, *n* (%)	137 (30)
Plasma levels of Aβ isoforms, pg/ml, mean (SD)	
Aβ_1–38_	19.4 (4.3)
Aβ_1–40_	186.1 (35.9)
Aβ_1–42_	56.3 (6.2)
MRI markers, ml, mean (SD)	
White matter volume	354.1 (85.4)
Gray matter volume	522.7 (55.8)
Intracranial volume	1126.2 (113.9)
Hippocampus volume	6.4 (0.9)

*Abbreviations*: , *APOE* Apolipoprotein, *Aβ* Amyloid-β, *MRI* Magnetic resonance imaging

Table 2 shows the cross-sectional analysis between Aβ levels and brain tissue volumes adjusted for age, sex, vascular risk factors, APOE-ε4 carrier status, intracranial volume, and other Aβ isoforms, when appropriate. Lower Aβ_1–38_, Aβ_1–40_, and Aβ_1–42_ levels and lower Aβ_1–40_/Aβ_1–42_ ratio were not associated with total brain, gray, and white matter volumes. A significant association was observed between lower plasma Aβ_1–42_ levels and smaller hippocampal volume (mean difference in hippocampal volume per SD decrease in Aβ_1–42_ levels, − 0.13; 95% CI, − 0.23 to − 0.04; *p* = 0.007).

**Table 2:** Association of amyloid-β levels with neurodegenerative markers

Plasma Aβ levels (per SD decrease)	Total brain volume, mean difference (95% CI),^a^ *p* value	Total gray matter volume, mean difference (95% CI),^a^ *p* value	Total white matter volume, mean difference (95% CI),^a^ *p* value	Total hippocampal volume, mean difference (95% CI),^a^ *p* value
Aβ_1–38_	0.03 (− 0.03; 0.08), *p* = 0.362	0.02 (− 0.10; 0.15), *p* = 0.715	0.01 (− 0.09; 0.12), *p* = 0.819	0.04 (− 0.11; 0.19), *p* = 0.582
Aβ_1–40_	− 0.04 (− 0.09; 0.02), *p* = 0.171	− 0.08 (− 0.21; 0.06), *p* = 0.248	0.01 (− 0.11; 0.12), *p* = 0.912	− 0.01 (− 0.17; 0.14), *p* = 0.869
Aβ_1–42_	0.00 (− 0.03; 0.04), *p* = 0.812	− 0.06 (− 0.14; 0.03), *p* = 0.174	0.04 (− 0.03; 0.12), *p* = 0.251	− 0.13 (− 0.23; − 0.04), *p* = 0.007
Aβ_1–40_/Aβ_1–42_ ratio	0.03 (− 0.02; 0.09) *p* = 0.231	0.06 (− 0.07; 0.19), *p* = 0.380	−0.00 (− 0.11; 0.11) *p* = 0.998	−0.10 (− 0.25; 0.05), *p* = 0.194

*Abbreviations*: *Aβ* amyloid-β, *SD* standard deviation, *CI* confidence interval

^a^ Adjusted for age, sex, mean arterial blood pressure, total cholesterol, diabetes, apolipoprotein ε4 carrier status, smoking, intracranial volume and other Aβ levels

During a mean follow-up of 14.8 years (SD, 4.9; range, 4.1–23.5 yr), 241 individuals died. In the same follow-up, 79 persons developed dementia. Of these 79 individuals with dementia, 64 were diagnosed with AD and 15 with vascular dementia. The interaction terms of Aβ levels with follow-up time in all models were nonsignificant (*p* value for interaction between Aβ_1–38_ and time, *p* = 0.832; between Aβ_1–40_ and time, *p* = 0.820; between Aβ_1–42_ and time, *p* = 0.998; and between Aβ_1–40_/Aβ_1–42_ ratio and time, *p* = 0.984). In the multivariable analysis, lower levels of Aβ_1–38_ and Aβ_1–42_ were associated with increased risk of dementia (HR for dementia per SD decrease in Aβ_1–38_, 1.33; 95% CI, 1.01–1.89; HR for dementia per SD decrease in Aβ_1–42_, 1.27; 95% CI, 1.02–1.58) (Table 3). No association was observed between Aβ_1–40_ and Aβ_1–40_/Aβ_1–42_ ratio and dementia. When the analysis was performed separately for dementia subtypes, lower levels of Aβ_1–38_ and Aβ_1–42_ were associated with increased risk of AD (HR for AD per SD decrease in Aβ_1–38_, 1.39; 95% CI, 1.00–2.16; HR for AD per SD decrease in Aβ_1–42_, 1.35; 95% CI, 1.05–1.75). The HRs for AD and vascular dementia were closely similar but insignificant in cases of vascular dementia (Table 3). The association between lower levels of Aβ and risk of dementia among APOE-ε4 carriers and noncarriers appeared similar, such that lower levels of Aβ_1–38_, Aβ_1–40_, and Aβ_1–42_ were associated with increased risk of dementia in both strata. A stronger association was observed for Aβ_1–38_ with increased risk of dementia among APOE-ε4 carriers (HR for dementia per SD decrease in Aβ_1–38_, 1.58; 95% CI, 1.01–2.89), whereas for Aβ_1–42_, this association was observed only among APOE-ε4 noncarriers (HR for dementia per SD decrease in Aβ_1–42_, 1.47; 95% CI, 1.09–1.99) (Table 4).

**Table 3:** Association of amyloid-β levels with incident dementia and its subtypes

Plasma Aβ levels (per SD decrease)	Incident dementia (*n* = 79) HR (95% CI)^a^	Alzheimer’s dementia (*n* = 64) HR (95% CI)^a^	Vascular dementia (*n* = 15) HR (95% CI)^a^
Aβ_1–38_	*1.33 (1.01–1.89)* ^*b*^	*1.39 (1.00–2.16)* ^*b*^	1.20 (0.49–2.96)
Aβ_1–40_	0.99 (0.69–1.43)	0.95 (0.60–1.49)	0.74 (0.31–1.79)
Aβ_1–42_	*1.27 (1.02–1.58)* ^*b*^	*1.35 (1.05–1.75)* ^*b*^	1.05 (0.61–1.79)
Aβ_1–40_/Aβ_1–42_ ratio	0.92 (0.64–1.32)	0.97 (0.64–1.49)	1.30 (0.54–3.11)

*Abbreviations*: *Aβ* amyloid-β, *SD* standard deviation, *HR* hazard ratio, *CI* confidence interval

^a^Adjusted for age, sex, education, mean arterial blood pressure, total cholesterol, diabetes, apolipoprotein ε4 carrier status, smoking and other Aβ levels

^*b*^Significant at *p* <0.05

**Table 4:** Association of amyloid-β levels with incident dementia in carriers and non-carriers of apolipoprotein ε4 allele

Plasma Aβ levels (per SD increase)	Incident dementia (*n* = 79) HR (95% CI)^a^	
	APOE-ε4 carriers (*n* = 35)	APOE-ε4 non-carriers (*n* = 44)
Aβ_1–38_	*1.58 (1.01–2.89)* ^*b*^	1.10 (0.66–1.83)
Aβ_1–40_	1.03 (0.58–1.83)	1.04 (0.64–1.71)
Aβ_1–42_	1.07 (0.76–1.52)	*1.47 (1.09–1.99)* ^*b*^
Aβ_1–40_/Aβ_1–42_ ratio	0.72 (0.41–1.26)	0.75 (0.45–1.16)

*Abbreviations*: *Aβ* amyloid-β, *SD* standard deviation, *HR* hazard ratio, *CI* confidence interval, *APOE* Apolipoprotein

^a^ Adjusted for age, sex, education, mean arterial blood pressure, total cholesterol, diabetes, smoking and other Aβ levels

^*b*^Significant at *p* <0.05

## Discussion

In this study, we showed that lower levels of plasma Aβ were not associated with preclinical markers of neurodegeneration (i.e., total gray matter and white matter volumes), except for Aβ_1–42_, which was associated with smaller hippocampal volume in elderly individuals. Individuals with lower levels of Aβ_1–38_ and Aβ_1–42_ had an independent increased risk of dementia, specifically AD. These findings suggest that Aβ_1–38_ and Aβ_1–42_ may be involved in different pathways leading to dementia.

Previous research has shown that higher plasma Aβ levels (1–40 and 1–42 peptides) at baseline were associated with cognitive dysfunction and faster cognitive decline, regardless of dementia status at follow-up, supporting the notion that plasma Aβ may induce a variety of brain pathologies (including cortical atrophy) earlier in life [27]. A recent study consisting of 100 participants also reported that higher plasma Aβ_1–42_ levels were associated with thinner temporal cortex in cognitively normal elderly persons [13]. However, apart from the latter study, no studies have yet combined the structural brain changes with plasma Aβ levels to assess the differences in cognitively normal elderly individuals. Moreover, the effects of novel Aβ_1–38_ in addition to Aβ_1–40_ and _1–42_ on cerebral atrophy and whether it is an important fluid biomarker for neurodegeneration and dementia has not been explored previously. Contrary to what we expected, our findings showed that lower plasma Aβ levels (including Aβ_1–38_) were not related to brain tissue volumes, except for hippocampal volume, where a significant association was observed between lower Aβ_1–42_ levels and smaller hippocampal volume in the elderly. The possible reason for the lack of association between Aβ_1–38_ and brain atrophy could be that plasma Aβ_1–38_ levels may reflect vascular disease in the brain rather than neurodegeneration. On one hand, this is supported by our previous study in which we showed that Aβ_1–38_ reflected microvascular damage in the brain and possibly induced adverse changes by inflammation, imbalance of oxygen free radicals, and apoptosis [22]. On the other hand, the association between Aβ_1–42_ and the hippocampus in the present study supports the notion that as Aβ_1–42_ starts to deposit in the brain after the age of 60 years, lower plasma Aβ levels that follow this deposition relate to hippocampal atrophy. A recent study has shown that accumulation of Aβ and tau pathologies in the brain were related to a decrease in hippocampal volume, including its critical subcompartments (i.e., CA1 and subiculum) in the earliest stages of AD prodromes [28]. Moreover, another study with healthy control individuals and persons with subjective cognitive complaints reported greater cortical thickness at intermediate levels of Aβ pathology (measured using CSF) [29]. By contrast, our data suggest that lower plasma Aβ levels (which are thought to indirectly reflect accumulation of amyloid in brain) do not necessarily correlate with universal volumetric decline in all structures (gray matter and white matter volumes), which is supported by some recent data [30].

Several lines of evidence suggest that Aβ levels in the CSF and plasma are in dynamic equilibrium with each other and that increased Aβ production in the brain gives rise to raised levels in the plasma [31]. It is further reported that as Aβ starts to deposit in the brain in the form of plaques, this in turn leads to lower plasma Aβ levels, which has been related to a higher risk of dementia [32]. Previous cross-sectional studies examining the association between Aβ levels and dementia have been mainly inconsistent [33–36]. It has been further reported that because plasma Aβ levels tend to change over the course of the dementia process, longitudinal studies are more useful in assessing the link between Aβ levels and risk of dementia in asymptomatic individuals [7]. Thus far, limited data have shown that plasma Aβ_1–42_ levels significantly decline in concentration in persons with newly diagnosed AD compared with individuals with prevalent AD and control subjects [12]. It is also suggested that plasma Aβ_1–42_ levels decline at an average rate of 12% per year among individuals with mild cognitive impairment [6]. Our results add to the previous reports by showing that lower levels of baseline plasma Aβ_1–38_, in addition to Aβ_1–40_ and Aβ_1–42_, were associated with a reduced risk of dementia. The association of Aβ_1–38_ and Aβ_1–42_ with dementia persisted when all the isoforms were added together in the model, indicating an independent link with increased dementia risk. These observations further support the findings that Aβ_1–38_ and Aβ_1–42_ may be generated independently by γ-secretase and that the production of these peptides is not coordinately regulated [16]. This was further confirmed in in vitro experiments involving γ-secretase modulators (sulindac sulfide), where Aβ_1–38_ levels were increased upon treatment with sulindac sulfide with no concurrent effect on Aβ_1–42_ levels, thus arguing against a precursor–product relationship [37, 38]. Though no association was observed between Aβ_1–38_ levels and neurodegenerative markers in this study, a link was still observed between lower levels of Aβ_1–38_ and increased risk of dementia. As mentioned before, the Aβ_1–38_ isoform is a marker of vascular pathology, and a higher level may indicate the activation of different inflammatory cascades (cytokines, cluster of differentiation 40 ligand, and tumor necrosis factor α) [22], which may increase the vulnerability to dementia, but such a relationship requires further clarification. Moreover, the relationship of plasma Aβ levels and brain amyloid deposition is suggested to be further complicated by dynamics of the blood-brain barrier and other possible sources of Aβ materials outside the central nervous system, including platelets and skeletal muscle cells [32].

With respect to the subtypes of dementia, similar associations were observed between plasma Aβ_1–38_ and Aβ_1–42_ levels with AD and vascular dementia. Though the significant results were observed only in cases of AD, this finding might be related to the fact that most of the individuals diagnosed with incident dementia had AD (72%). Also, in an elderly population, mixed pathology is commonly observed, which might explain the similar estimates for AD and vascular dementia. Moreover, the association of lower levels of Aβ_1–38_ with increased risk of dementia was more significant in carriers of the APOE-ε4 allele than in noncarriers. Though an opposite link was observed between Aβ_1–42_ and increased dementia risk in APOE-ε4 noncarriers, the similarity in the direction of effect estimates further suggests a role of mixed pathology in the development of dementia, regardless of APOE carrier status.

Strengths of our study include its prospective design, the population-based setting, volumetric quantification of the brain tissues, and virtually complete follow-up for dementia in the older cohort. There are some potential limitations of our study. First, because plasma samples were collected after MRI acquisition with a 3-year time window, the effect estimates calculated in this study may represent over- or underestimations. Second, plasma Aβ levels provide an indirect measure of brain-specific Aβ pathology, and an in vivo analysis of brain-specific Aβ burden (such as with amyloid positron emission tomography) could allow more accurate measures of Aβ burden. Finally, we lacked repeated measurement of plasma Aβ concentrations, which limits its ability to better estimate the trajectory of plasma levels over time in relation to risk of dementia. However, this could be a subject for further studies.

## Conclusions

This study provides evidence that lower plasma Aβ levels, specifically Aβ_1–38_ and Aβ_1–42_, are associated with increased risk of dementia, specifically AD. Future studies should examine whether inclusion of novel plasma Aβ_1–38_ levels as an additional biomarker can provide further information on risk of developing dementia and AD dementia.

## Abbreviations

AD: Alzheimer’s disease
APOE: Apolipoprotein E
Aβ: Amyloid-β
CSF: Cerebrospinal fluid
EDTA: Ethylenediaminetetraacetic acid
GMS: Geriatric Mental State Schedule
HASTE: Half-Fourier-acquired single-shot turbo spin echo
MMSE: Mini Mental State Examination
MRI: Magnetic resonance imaging
QC: Quality control
